# Impact of *Ficus deltoidea* Aqueous Extract on Maternal Hepatic Drug Metabolism and Foetal Development in Rats

**DOI:** 10.3390/plants14233623

**Published:** 2025-11-28

**Authors:** Hussin Muhammad, Nik Aina Syazana Nik Zainuddin, Wan Mazlina Md Saad, Maizatul Hasyima Omar, Ezarul Faradianna Lokman

**Affiliations:** 1 Toxicology and Pharmacology Unit, Herbal Medicine Research Centre, Institute for Medical Research, National Institutes of Health, Ministry of Health, Shah Alam Selangor 40170, Malaysia; nikaina@moh.gov.my; 2 Centre of Medical Laboratory Technology, Faculty of Health Science, Universiti Teknologi MARA, Puncak Alam Campus, Puncak Alam Selangor 42300, Malaysia; wanmaz755@uitm.edu.my; 3 Phytochemistry Unit, Herbal Medicine Research Centre, Institute for Medical Research, National Institutes of Health, Ministry of Health, Shah Alam Selangor 40170, Malaysia; maizatul.hasyima@moh.gov.my; 4 Endocrine and Metabolic Unit, Nutrition, Metabolism and Cardiovascular Research Centre, Institute for Medical Research, National Institutes of Health, Ministry of Health, Shah Alam Selangor 40170, Malaysia; fara@moh.gov.my

**Keywords:** *Ficus deltoidea*, skeletal, maternal, rats, drug metabolism

## Abstract

The present study aimed to assess the potential maternal toxicity of *Ficus deltoidea* var. *kunstleri* aqueous extract in pregnant rats, along with its impact on maternal hepatic drug metabolism and foetal skeletal development. Pregnant rats were divided into five groups and orally administered varying doses of *F. deltoidea* aqueous extract (0, 250, 500, 1000, and 2000 mg/kg body weight) from gestation day 6 to 20. Throughout the administration period, clinical observations, body weight, and food and water intake were monitored. On gestation day 21, the pregnant rats were sacrificed, and their vital organs and foetuses were collected for analysis. Gene expression related to hepatic drug metabolism was evaluated using the RT2 Profiler™ PCR array. Foetal external morphology was examined for abnormalities, and skeletal structures were stained with Alizarin Red to assess the effects of *F. deltoidea* aqueous extract on bone ossification during organogenesis. No maternal toxicity was observed, except for a significant increase in liver weight in the treated groups (*p* < 0.05). Analysis of 84 genes revealed significant changes in 15, 4, and 11 genes in the 250, 500, and 2000 mg/kg body weight groups, respectively. Notably, *Gpx5* and *Pkm*, both phase II metabolising enzyme genes were downregulated in a dose-dependent manner. Despite some skeletal variations, the extract did not induce foetal external malformations or skeletal abnormalities. The significant increase in maternal liver weight, together with the downregulation of *Gpx5* and *Pkm*, suggests an adaptive hepatic response to the extract rather than an adverse effect. These findings also suggest that *F. deltoidea* var. *kunstleri* aqueous extract does not cause embryo toxicity, foetal growth retardation, or developmental malformations, particularly in skeletal formation. The developmental no-observed-adverse-effect level (NOAEL) was determined to be >2000 mg/kg/day via oral administration. Further research is warranted to explore the synergistic interactions of genes involved in hepatic drug metabolism in response to the extract.

## 1. Introduction

Approximately 70–80% of the global population, particularly in developing countries, relies on plant-based remedies as their primary form of healthcare [1]. In Malaysia, *Ficus deltoidea* var. *kunstleri* (King) Corner (Plant of the World online), commonly known as mas cotek, has been traditionally used to treat various ailments. The powdered roots and leaves of *F. deltoidei* Jack have long been utilised in ethnomedicine for managing diabetes, wounds, rheumatism, toothaches, headaches, colds, sore throats, and postnatal recovery, as well as for regulating the menstrual cycle [2].

Research on its pharmacological properties has revealed that *F. deltoidea* exhibits antioxidant, anti-inflammatory, and anti-tumour activities [3,4,5,6,7]. These therapeutic effects are attributed to the bioactive compounds present in different parts of the plant, including the roots, bark, leaves, and figs [8]. Phytochemical analysis has identified flavonoids, isovitexin, vitexin, proanthocyanidins, flavan-3-ol monomers, and flavone glycosides as key chemical constituents of *F. deltoidea* [9].

Due to the common belief that herbal medicines are derived from natural sources and are therefore safe, many pregnant women consume herbal-based products to alleviate discomfort, facilitate labour, and enhance overall health. Traditionally, postpartum women use leaf decoctions to help contract the womb, regulate menstrual cycles, and improve blood circulation [10]. Among the various *F. deltoidea* variants, *F. deltoidea* var. *deltoidea* and *F. deltoidea* var. *angustifolia* var. *angustifolia* have been shown to stimulate uterine contractions [7].

Preclinical studies using animal models have demonstrated the safety of *F. deltoidea* in genotoxicity assessments, as well as in single and repeated exposure studies [11]. While no cases of toxicity have been reported in humans, caution is necessary, as the plant may pose potential risks to both mother and foetus. Therefore, this study aimed to evaluate maternal toxicity and skeletal malformations during prenatal development to assess the safety of *F. deltoidea* var. *kunstleri* aqueous extract during pregnancy.

## 2. Results

### 2.1. Phytochemical Content of F. deltoidea Var Kunstleri Aqueous Extract

**Figure 1:**
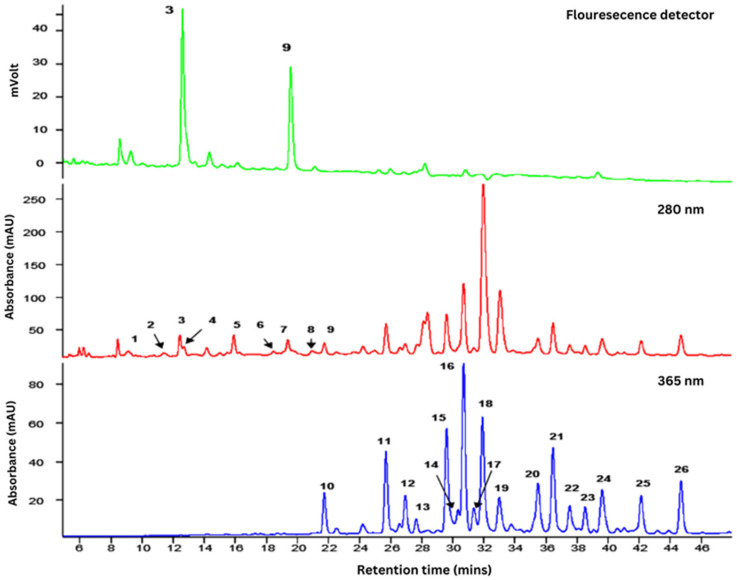
Mass spectral characteristics and identity of phenolics present in *F. deltoidea* var. *kunstleri* analysed by HPLC coupled to tandem mass spectrometry.

The aqueous extract of *F. deltoidea* revealed twenty flavonoids, as shown in Table 1 and Figure 1. (+)-Catechin (peak 3) and (−)-Epicatechin (peak 6) were detected using fluorescence detector at the (λEX/λEM) 280/315 nm wavelengths [12].

### 2.2. Maternal Mortality, Clinical Observations, and Food and Water Intake

**Figure 2:**
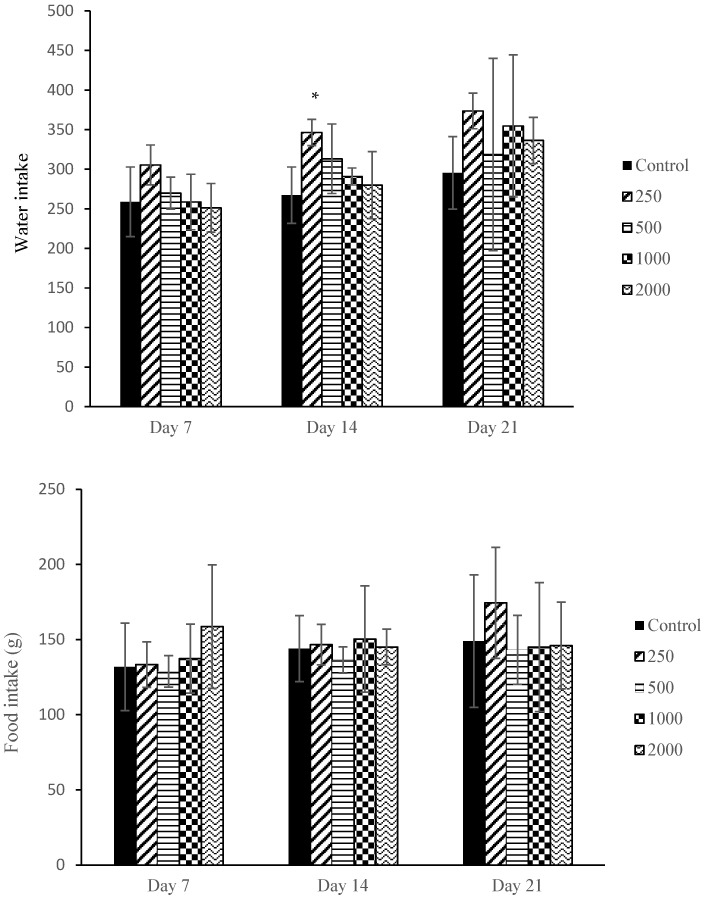
Water and food intake of dams after orally treated with different concentrations of *F. deltoidea* var*. kunstleri* aqueous extract on GD6-20. Each value represents the mean ± SD (*n* = 5). * *p* value < 0.05 compared to control.

No maternal deaths were registered and no adverse clinical signs were displayed such as piloerection, vaginal bleeding, diarrhoea, alteration in locomotion, dull fur, emaciation, soft stool or urination until the scheduled necropsy. No significant changes were observed in the maternal food consumption. A significant increase in water consumption was only observed in the 250 mg/kg bw group on day 14, as shown in Figure 2.

### 2.3. Maternal Body Weight and Body Weight Gain

**Figure 3:**
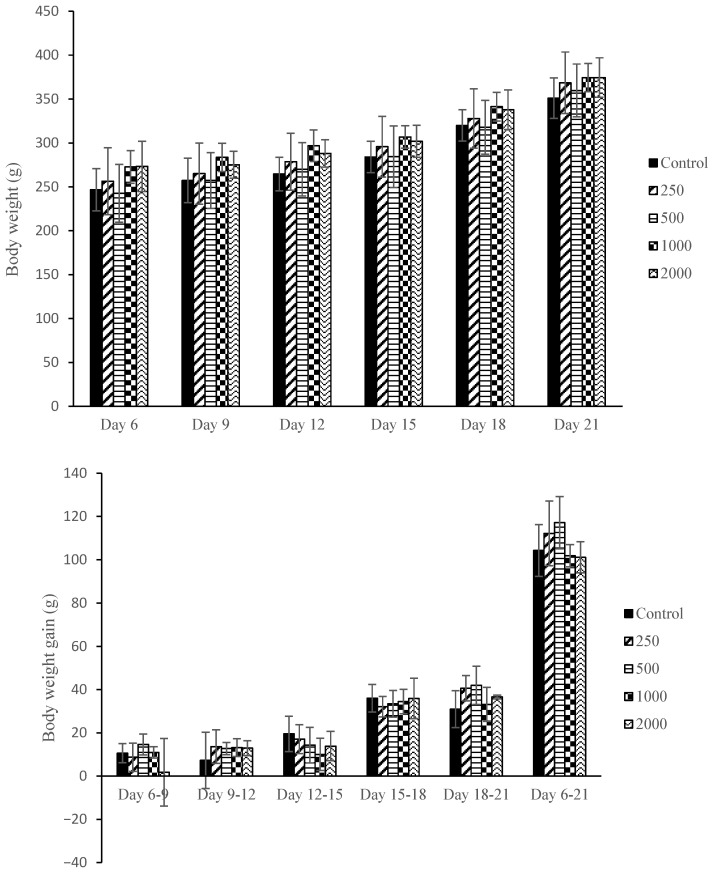
Body weight and body weight gain of dams after orally treated with different concentrations of *F. deltoidea* var*. kunstleri* aqueous extract on GD6-20. Each value represents the mean ± SD (*n* = 5). Statistical analysis (ANOVA) did not show any significant differences among groups.

Figure 3 shows the effect of *F. deltoidea* var. *kunstleri* aqueous extract administration on the body weight and body weight gain of pregnant rats on the following gestation days (GDs): GD6-9, GD9-12, GD12-15, GD15-18, and GD18-20. No significant changes were observed in all treated groups compared to the control (*p* > 0.05).

### 2.4. Maternal Visceral Organs Examination

There were no abnormal findings on the maternal organs observed in the liver, heart, lungs, kidneys, and ovaries. No significant changes in the relative organ weight were observed in all groups except a significant increase (*p* < 0.05) in the liver compared to the control, as shown in Table 2.

### 2.5. Gene Expression Analysis of Hepatic Drug Metabolism and Detoxification Pathways

In this study, gene expression profiling focused on key hepatic genes involved in drug metabolism, biotransformation, xenobiotic transport, oxidative stress regulation, and lipid homeostasis. These included phase I enzymes (*Cyp19a1*, *Cyp27b1*, *Cyb5r3*), phase II conjugating enzymes (*Gstt1*, *Gstm1*), drug efflux transporters (*Abcb1a*, *Abcb4*), antioxidant-related genes (*Gpx3*, *Gpx5*, *Blvra*), and metabolic regulators (*Pkm*, *Gckr*, *Faah*, *Gad1*). These genes were selected because they represent major pathways potentially influenced by herbal constituents and are widely recognised markers of the hepatic adaptive response to xenobiotics. Understanding their modulation provides insight into how *F. deltoidea* var. *kunstleri* aqueous extract may affect maternal liver metabolism during gestation.

**Figure 4:**
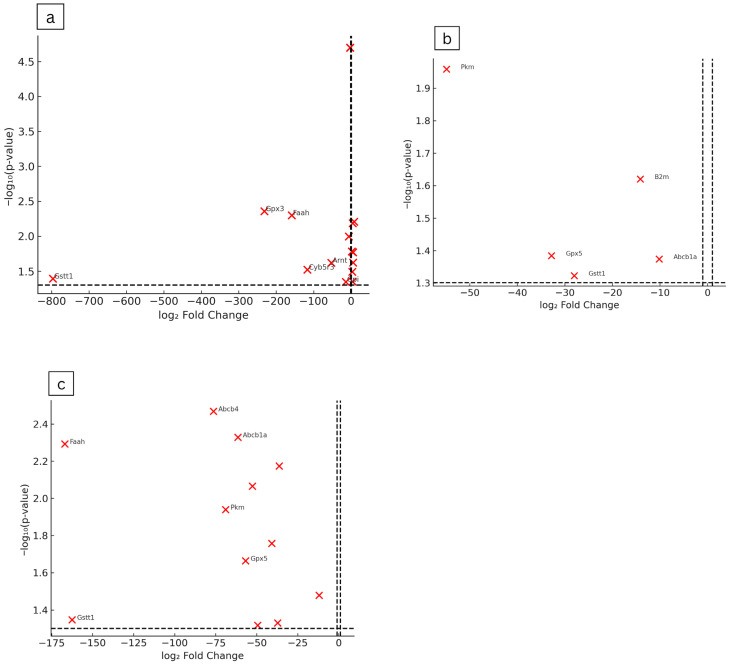
Volcano plots showing differential expression of maternal hepatic genes following exposure to *F. deltoidea* var. *kunstleri* aqueous extract at (**a**) 250 mg/kg, (**b**) 500 mg/kg, and (**c**) 2000 mg/kg. Each point represents 1 of the 84 genes assessed in the predefined drug metabolism and toxicity panel. The *x*-axis displays the log_2_ fold change relative to the untreated control group, while the *y*-axis shows the −log_10_(*p*) value. The dotted line on 0-line acts as the baseline for interpreting direction of regulation. Vertical dashed lines indicate the ±1 log_2_ fold-change thresholds, and the horizontal dashed line represents the statistical significance threshold (−log_10_(0.05)). Genes demonstrating the largest magnitude changes are labelled. These plots summarise the dose-dependent transcriptional response in maternal liver tissue and highlight key modulated genes, including *Gstt1*, *Faah*, *Gpx3*, *Pkm*, and *Abcb4*.

**Figure 5:**
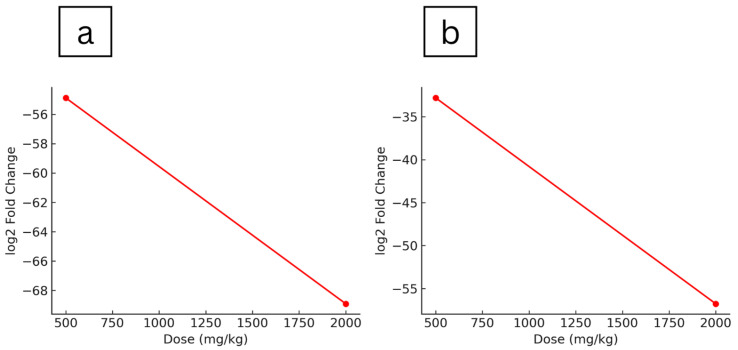
Dose–response mini-plots for (**a**) *Pkm* and (**b**) *Gpx5* in maternal liver following treatment with *F. deltoidea* var. *kunstleri* aqueous extract. Both genes showed progressive, dose-dependent downregulation from 500 to 2000 mg/kg, indicating suppression of hepatic metabolic and antioxidant pathways with increasing extract exposure.

The hepatic gene expression profiles of maternal rats treated with *Ficus deltoidea* var. *kunstleri* aqueous extract during the gestational period were evaluated (Table 3). Significant upregulation occurred only in the lowest-dose group (250 mg/kg), with modest (<10-fold) increases in genes such as *Abcb4*, *Ahr*, *Gckr*, *Gstm1*, and *Nat1*. At the same dose (250 mg/kg), several genes were markedly downregulated, including *Gstt1* (−796.33, *p* = 0.0405), *Gpx3* (−231.63, *p* = 0.0044), *Faah* (−158.27, *p* = 0.005), *Cyb5r3* (−116.62, *p* = 0.0300), *Abcb1a* (−61.45, *p* = 0.0047), *Arnt* (−52.80, *p* = 0.0240), and *Cyp27b1* (−5.95, *p* = 0.0101), with smaller reductions in *Cyp19a1*, *Gad1*, and *Snn* (−2.59, *p* = 0.00002). In the highest-dose group (2000 mg/kg), downregulated genes included *Faah* (−166.92, *p* = 0.0510), *Gstt1* (−162.61, *p* = 0.0449), *Abcb4* (−76.40, *p* = 0.0340), *Pkm* (−68.91, *p* = 0.0115), *Abcb1a* (−61.45, *p* = 0.0047), *Gpx5* (−56.78, *p* = 0.0217), *Ces2c* (−49.43, *p* = 0.0481), *Arnt* (−40.75, *p* = 0.0175), *Cyp27b1* (−36.33, *p* = 0.0670), *Gpi* (−14.07, *p* = 0.0450), and *Blvra* (−12.00, *p* = 0.0320). At 500 mg/kg, genes showing downregulation included *Pkm* (−54.88, *p* = 0.0110), *Gpx5* (−32.81, *p* = 0.0413), *Gstt1* (−28.04, *p* = 0.0476), and *Abcb1a* (−10.16, *p* = 0.0423). Volcano plot analysis showed dose-dependent alterations in maternal hepatic gene expression following *Ficus deltoidea* var. *kunstleri* treatment. Several genes exceeded the significance thresholds (±1 log_2_ fold change, −log_10_(*p*) ≥ 1.30), with the largest changes observed for *Gstt1*, *Faah*, *Gpx3*, *Pkm*, and *Abcb4*. These genes were predominantly downregulated at higher doses, indicating a concentration-related suppression of selected metabolic and oxidative stress–related pathways (Figure 4). Overall, gene upregulation was observed only at the lowest dose, while *Gpx5* and *Pkm* demonstrated a clear dose-dependent downregulation pattern, indicating progressive suppression of these metabolic genes with increasing extract concentration (Figure 5).

### 2.6. Foetal Examination

**Figure 6:**
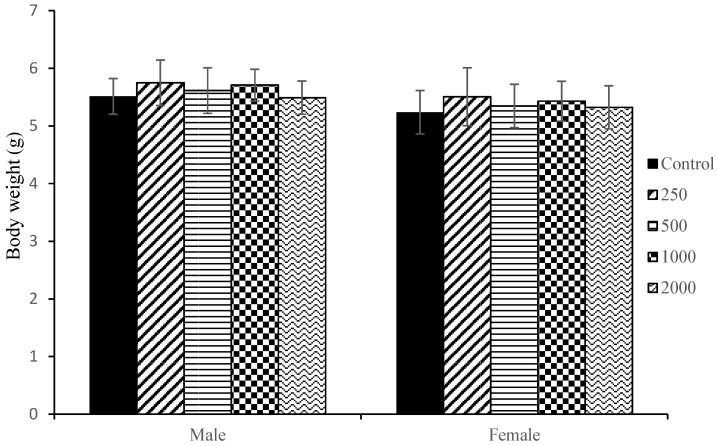
Foetal body weight after dams were orally treated with different concentrations of *F. deltoidea* var. *kunstleri* aqueous extract on GD6-20. Each value represents the mean ± SD (*n* = 5). Statistical analysis (ANOVA) did not show any differences among groups.

No significant foetal gross dysmorphology or foetal death was observed in any treatment groups when compared to the control. The mean foetal body weight for both sexes was almost similar and comparable to the control group though male body weight was slightly higher than female weight (Figure 6).

### 2.7. Skeletal Examination of Foetuses

Skeletal abnormalities in foetal rats were assessed in a total of 150 foetuses, with the findings summarised in Table 4. The skulls were examined for shape and degree of ossification. A significantly higher number of additional ossification centres in the os interparietalis bone was observed in foetuses from the 500, 1000, and 2000 mg/kg body weight treatment groups compared to the control (*p* < 0.05). Additionally, a significant increase in incomplete ossification of the frontal bone was detected in foetuses exposed to 500 and 1000 mg/kg body weight. The absence of the hyoid body was more frequently observed in foetuses treated with 250 and 1000 mg/kg body weight of *F. deltoidea* var. *kunstleri* aqueous extract. Although this difference was statistically significant compared to the control, the effect did not follow a dose-dependent pattern.

The sternum was examined by assessing five sternebrae (1, 2, 3, 4, and 5) along with the xiphisternum. A significant increase in incomplete ossification of the xiphisternum was observed in foetuses from the highest dose group (2000 mg/kg body weight). In the vertebral column, a notable number of dumbbell-shaped thoracic vertebrae were identified in foetuses treated with 500 and 1000 mg/kg body weight of the extract. Other skeletal variations were observed in some foetuses across the groups; however, these changes were not statistically significant and were comparable to those in the control group.

The development of long bones in the forelimbs, including metacarpals, proximal, middle, and distal phalanges, as well as the clavicle, scapula, humerus, radius, and ulna, was assessed by evaluating their number, shape, and degree of ossification. Poor ossification of the phalanges was observed across all groups; however, the difference was not statistically significant compared to the control group (*p* > 0.05).

**Figure 7:**
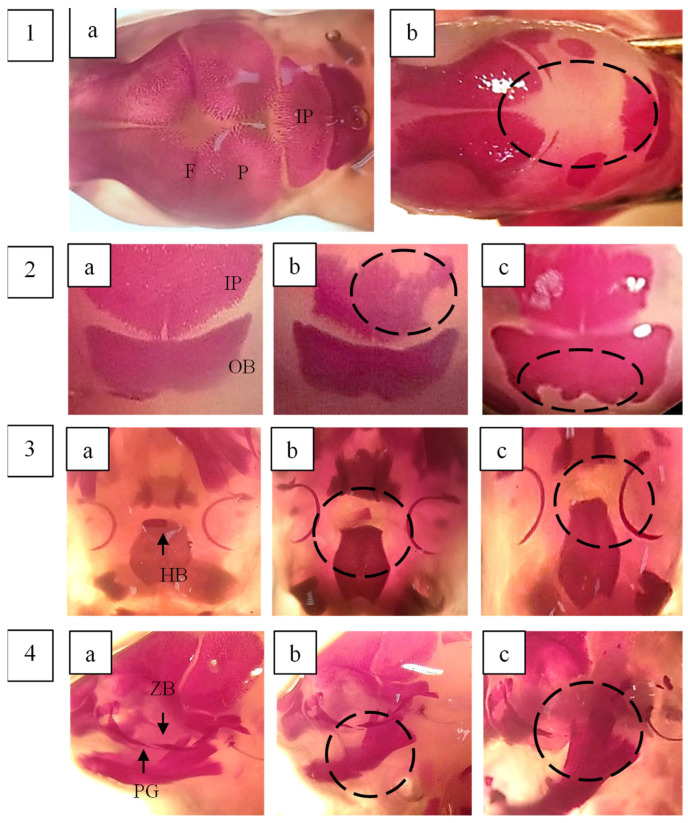
Pictures represent some of the skeletal changes observed in foetal rats: (**a**) control; (**b**,**c**) treament. (**1**)—The frontale (F), parietal (P), and interparietal (IP) bones of foetal rat. (**a**) Normal F, P, and IP; (**b**) incomplete ossification of P and both sides of IP. (**2**)—(**a**) Normal IP and occipital bone (OB), (**b**) incomplete ossicifation of IP, (**c**) incomplete ossification of OB. (**3**)—(**a**) Normal hyoid body (HB), (**b**) incomplete ossification of HB, (**c**) unossified of HB. (**4**)—(**a**) Normal processus jugalis of maxilla (PG) and zygomatic bones (ZB), (**b**) incomplete ossification of PG, (**c**) unossified of ZB. The foetuses were collected by caesarean hysterectomy on GD21 and stained with Alizarin Red S. Bone deformities are indicated by circles. Statistical analysis (one-way ANOVA) did not show any differences between control and treated groups (*p* > 0.05). Magnification: 125×.

**Figure 8:**
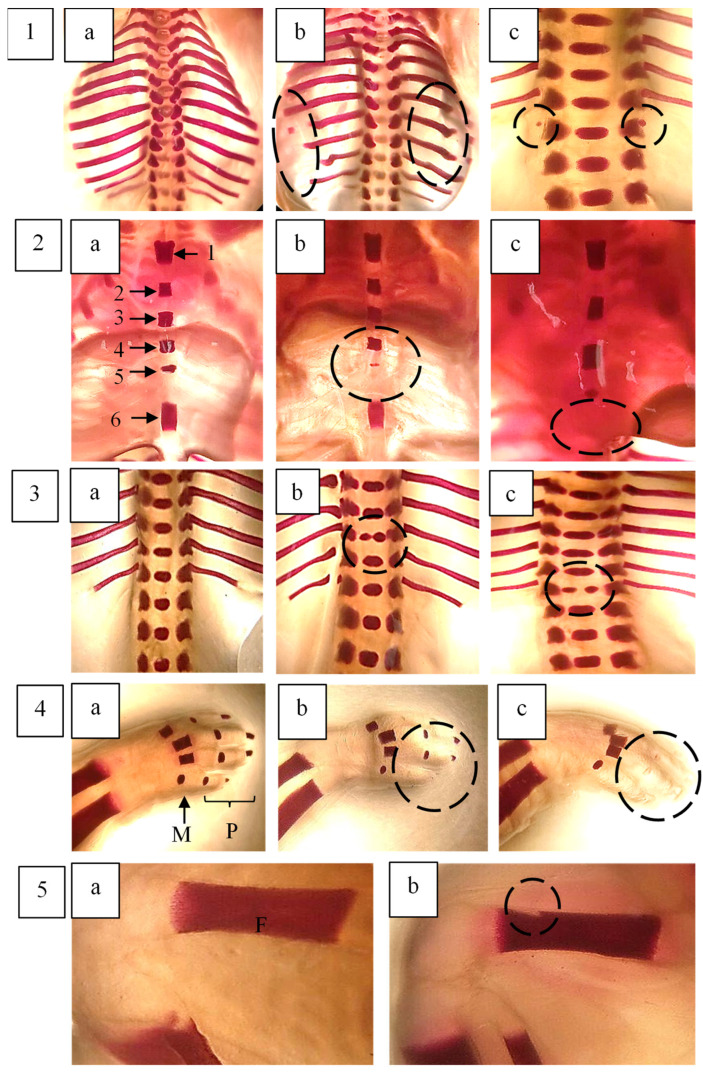
Pictures represent some of the skeletal changes observed in foetal rats: (**a**) control; (**b**,**c**) treament. (**1**)—The ribs of foetal rat. (**a**) Normal ribs; (**b**) malformed wavy ribs and incomplete ossification; (**c**) extra rudimentary 14th rib on both sides. (**2**)—(**a**) Normal sternerbrae (ossification centre 1,2,3,4,5,6 (xiphisternum)), (**b**) incomplete ossicifation of ossification centre 5, (**c**) absent ossification centre 6. (**3**)—(**a**) Normal thoracic verterbrae and central nuclei, (**b**) dumbbell-shaped thoracic centre (11th), (**c**) bipartite thoracic centre (13th). (**4**)—(**a**) Normal fingers—metacarpus (M) and phalanx (P); (**b**) incomplete ossification of P; (**c**) unossified P. (**5**)—(**a**) Normal femur (F); (**b**) misshapened F. The foetuses were collected by caesarean hysterectomy on GD21 and stained with Alizarin Red S. Bone deformities are indicated by circles. Statistical analysis (one-way ANOVA) did not show any differences between control and treated groups (*p* > 0.05). Magnification: 125×.

Hindlimbs were examined based on their shape, size, and degree of ossification. A significant effect of *F. deltoidea* var. *kunstleri* aqueous extract on the femur (misshapen) was noted in both the control and treated groups. Images illustrating some of these skeletal changes are presented in Figure 7 and Figure 8.

## 3. Discussion

Normal foetal development is closely linked to maternal health and is influenced by a complex interplay of genetic, immunological, endocrinological, nutritional, vascular, and environmental factors. Any disruption to these factors can interfere with normal growth and development [13]. Exposure to xenobiotics during pregnancy may impact both foetal development and maternal well-being. Typically, maternal toxicity is assessed through clinical signs, changes in food and water consumption, mortality, body weight gain, and histopathological alterations [14].

In this study, the administration of *F. deltoidea* var. *kunstleri* aqueous extract at doses up to 2000 mg/kg body weight did not result in any behavioural changes, alterations in body weight, or deviations in body weight gain among pregnant rats throughout the gestation period. However, a significant increase in liver weight was observed in all treated groups. Similar findings have been reported in studies on *Ficus asperifolia* aqueous and methanol extracts, where female rats treated with 100 and 500 mg/kg body weight, respectively, exhibited increased liver weight after 21 days of administration [15].

The liver plays a crucial role in detoxifying xenobiotics and protecting against chemical toxicity. Xenobiotic-induced liver injury is a significant cause of hepatic disease and can potentially lead to liver failure. Various static and dynamic biomarkers are commonly used to assess liver function and detect potential liver damage [16]. During pregnancy, exposure to xenobiotics may lead to embryotoxicity and teratogenicity, particularly at high doses nearing toxic levels. Some of these xenobiotics may be well tolerated by the mother but can still pose risks to foetal development.

During normal pregnancy, both human and rat livers undergo metabolic adaptations to meet the increased energy demands of the developing foetus and to facilitate the detoxification of foetal metabolites [17]. These metabolic changes involve key parameters such as insulin-like growth factor (IGF), growth hormone, placental lactogen, and bile acids, often leading to liver enlargement. A previous study on diabetic-induced rats administered with *F. deltoidea* methanol leaf extract at 1000 mg/kg body weight reported a significant increase in hepatic insulin production [18]. Additionally, Ham et al. (2020) [19] found that *F. deltoidea* treatment in hypercholesterolemic-induced rats reduced cholesterol absorption by enhancing bile acid excretion. Based on these findings, the observed increase in liver weight in our study may be attributed to elevated bile acid production stimulated by *F. deltoidea* var. *kunstleri* extract in pregnant rats. Furthermore, the increased liver weight observed in all treated groups, in the absence of overt toxicity or foetal malformations, may represent an adaptive hepatic response linked to the modulation of xenobiotic-metabolising pathways. The downregulation of *Gpx5* and *Pkm* observed in our present study, which participate in antioxidant defence and glycolytic energy balance within phase II metabolism, indicates a metabolic adjustment to sustained phytochemical exposure. Such changes are characteristic of enzyme adaptation, which is a reversible physiological process rather than hepatocellular injury. Although a significant, uniform increase in maternal liver weight was observed, the present study did not include histopathological or biochemical assessments of hepatic function, which limits the ability to definitively exclude hepatocellular injury. Nonetheless, similar findings have been reported in pregnant rats, where physiological hepatomegaly occurs due to enhanced hepatic metabolism and hormonal adaptation during gestation particularly after implantation and parturation [20].

Additionally, studies on rats have shown that pregnancy influences hepatic drug metabolism activity, although the exact mechanisms remain unclear. To explore this, we analysed the gene expression profile related to drug metabolism to assess its association with *F. deltoidea* var. *kunstleri* aqueous extract administration. It is well established that the metabolism of most xenobiotics is catalysed by hepatic cytochrome P450 enzymes, including *Cyp1*, *Cyp2*, and *Cyp3* isoenzymes [21]. In fact, the expression levels of these genes are typically downregulated during pregnancy [22].

Interestingly, our findings align with these reports, as we observed significant downregulation of *Cyp27b1* and *Cyp4b1* in animals treated with the highest concentration of *F. deltoidea* extract. A similar pattern was noted for pyruvate kinase M1/2 (*Pkm*) and glutathione peroxidase 5 in the 500 and 2000 mg/kg body weight groups—genes known for their protective roles against liver injury [23,24]. These results suggest that *F. deltoidea* var. *kunstleri* extract may influence hepatic drug metabolism through complex gene interactions, highlighting the need for further investigation into its mechanistic effects.

Foetal size serves as a crucial indicator of developmental toxicity, with any reduction potentially linked to the toxic effects of xenobiotics [25]. In this study, foetal body weight was measured on the day of parturition. The analysis showed no significant differences in foetal weight between the treatment groups and the control, indicating that *F. deltoidea* var. *kunstleri* aqueous extract did not adversely impact foetal growth.

Previous research has demonstrated that various factors, including intrauterine growth rates, nutritional status, foetal sex, genetic influences, maternal metabolism, placental vascularisation, and maternal weight, can affect foetal body weight [26,27]. To further assess potential developmental delays, we also evaluated foetal skeletal development as an additional parameter.

During pregnancy, the maternal body undergoes significant physiological adjustments to maintain calcium homeostasis and ensure proper foetal skeletal development. A substantial amount of calcium is transferred to the foetus through the placenta, particularly in late pregnancy, to support skeletal mineralisation [28]. In rats, foetal calcium accumulation remains below 0.5 mg during the first 17 days of gestation but rises sharply to 12 mg in the final days [29]. However, this process can be disrupted by maternal and/or embryotoxic compounds.

Bone alterations can be explained through three key factors: lack of homogeneity, reduced density, and decreased stained area. Indicators of delayed ossification in the rat foetal skeleton include incomplete, poorly calcified, or unossified bones [30]. In this study, skeletal abnormalities observed in the offspring of *F. deltoidea* var. *kunstleri* aqueous extract-treated rats—such as incomplete ossification of the os frontale, occipitale, interparietale, xiphisternum, and bipartite-shaped thoracic vertebra centra—are generally categorised as variations [31]. These variations are unlikely to impact survival or overall health, as normal skeletal development is expected to continue. However, the absence of the hyoid body has been classified as a malformation, though the changes observed were not dose-dependent.

Beyond calcium, other essential minerals such as phosphorus, magnesium, and zinc play crucial roles in foetal growth and bone development. However, data linking the low intake of these minerals to reduced foetal bone mineralisation remain limited. Current studies on *F. deltoidea* var. *angustifolia* and *F. deltoidea* var. *deltoidea* have identified the presence of magnesium, manganese, iron, and zinc [32]. Previous studies have also reported consistent differences in flavonoid content among the respective varieties [33]. Therefore, further investigation is needed to determine the contribution of these micronutrients and different metabolites to foetal skeletal development.

Several studies have established a link between maternal toxicity and foetal skeletal malformations, as well as reduced foetal body weight [34,35]. However, our findings indicate that the administration of *F. deltoidea* var. *kunstleri* aqueous extract did not induce maternal toxicity or negatively impact skeletal development in rat foetuses. These results suggest that *F. deltoidea* var*. kunstleri* aqueous extract is safe for use in animal models during pregnancy.

## 4. Materials and Methods

### 4.1. Chemicals

Glycerol and potassium hydroxide purchased from Merck, Chemical Germany (Darmstadt, Germany). Diethyl ether was purchased from Fisher (Waltham, MA, USA). Alizarin Red S from Sigma Aldrich (Burlington, MA, USA).

### 4.2. F. deltoidea Aqueous Extraction Preparation

Fresh leaves of *F. deltoidei* var. *kunstleri* were obtained from a local farmer in Pekan, Pahang, Malaysia. The plant material was authenticated, and a voucher specimen (SK3024/16) was deposited at the Herbarium, Institute of Bioscience, Universiti Putra Malaysia (UPM), Malaysia. The leaves were then dried in a hot-air oven at 45–50 °C and subsequently ground into a fine powder.

A 100 mg portion of the powdered of *F. deltoidea* was dissolved in 10 mL of methanol–water (50%) to obtain a final concentration of 10 mg/mL. The solution was then centrifuged at 10,000× *g* for 5 min at 4 °C and filtered using a 0.4 µm Whatman No.1 filter. The prepared samples were analysed using HPLC with absorbance, fluorescence, and mass spectrometric detection. The HPLC system utilised a Surveyor gradient system (Thermo-Finnigan, San Jose, CA, USA) comprising a pumping system, autosampler, and degasser, coupled with a photodiode array (PDA) detector scanning from 200 to 700 nm, controlled by Xcalibur software version 1.3. Chromatographic separation was performed using a MAX-RP 4 µm, 250 mm × 4.6 mm C12 reverse-phase column (Phenomenex, Torrance, CA, USA) maintained at 40 °C. The mobile phase consisted of a gradient elution from 15% to 50% methanol in water containing 0.1% formic acid over 60 min at a flow rate of 1.0 mL/min. Following passage through the PDA and fluorescence detectors, the column eluate was split, with 20% directed to an LCQ Duo mass spectrometer (Thermo-Finnigan) equipped with an electrospray interface operating in full scan data-dependent MS/MS mode (150–1000 amu). (+)-Catechin and (−)-Epicatechin were identified using a fluorescence (FL) detector (Jasco FP-920, Tokyo, Japan) at excitation/emission wavelengths of 280/315 nm.

### 4.3. Animal

Healthy virgin female and confirmed fertile male Sprague Dawley rats, weighing between 180 and 250 g, were obtained from the Laboratory Animal Resource Unit, Special Resource Centre (SRC), Institute for Medical Research, Malaysia. The animals were housed in polypropylene cages lined with corn cob bedding under controlled conditions: a temperature of 20 ± 2 °C, humidity levels of 40–60%, and a 12 h light–dark cycle. They were acclimatised for one week before the study commenced. A commercial rat diet (Specialty Feeds, Australia) and water were provided ad libitum. This study was conducted in compliance with the Animal and Research Ethics Act and was approved by the Ministry of Health Malaysia (ACUC No: ACUC/KKM/02(2/2017)).

### 4.4. Study Design

Female Sprague Dawley rats in the pro-oestrous phase were introduced into the male’s cage in the late morning and remained there for 24 h. Mating was confirmed by the presence of sperm in the vaginal smear, marking gestation day 0 (GD0). Pregnant rats were then randomly assigned to five groups: control (distilled water) and treatment groups receiving 250, 500, 1000, or 2000 mg/kg body weight of *F. deltoidea* aqueous extract (*n* = 5/group). The extract was administered via oral gavage from gestation day 6 to day 20. Rats were observed once daily for 60 min to monitor behavioural changes and clinical signs of toxicity following administration. Maternal body weight was recorded daily, while food and water intake were measured and documented weekly. On GD21, rats were euthanised and caesarean section was performed. Foetuses were collected, weighed and examined for any abnormalities.

### 4.5. Skeletal Examination of Foetuses

In this study, foetuses from each litter were coded before selection. Half of the foetuses from each dam were randomly chosen using a random number generator to avoid selection bias. Both male and female foetuses were included proportionally to the litter sex ratio, ensuring no systematic sex bias in the subset examined. Selected foetuses were fixed in 10% neutral-buffered formalin for at least two weeks before processing. They were then washed and soaked in tap water for 24 h, bisected at the abdominal region, and had their internal organs removed. The samples were cleared in a 1:4 diethyl ether–methanol solution for one week, washed, and restained with 0.3% Alizarin Red S in 40 mL of KOH for one week. After staining, soft tissues were cleared, and specimens were transferred to a 1:1 glycerin–ethanol mixture for 24 h and preserved in 100% glycerol. The skeletal structures were examined under a stereo microscope (Motic, San Francisco, CA, USA) and scored by an investigator blinded to the treatment groups, using anonymised sample codes to minimise subjective bias during morphological assessment. Six key skeletal regions like skull, sternum, ribs, vertebral column, forelimbs, and hindlimbs were evaluated for ossification status and variations. The skeletal scoring was conducted by an investigator blinded to the treatment groups, using anonymised sample codes to minimise subjective bias during morphological assessment.

### 4.6. Gene Expression Profiles of Maternal Rat Liver

Liver tissues (30 mg) from different treatment groups (control, 250, 500, and 2000 mg/kg body weight) were weighed, and 10% β-mercaptoethanol in RLT buffer was added. The tissues were then cut into small pieces and homogenised using a tissue ruptor (50–60 Hz, 230 V) (Qiagen, Germantown, MD, USA) with disposable probes. The homogenised samples were processed to extract RNA using the RNeasy Mini Kit (Qiagen, USA). The purity and concentration of the extracted RNA were assessed using a Nanodrop (Thermo Fisher Scientific, Waltham, MA, USA), ensuring that the absorbance ratio at 260/280 nm ranged between 1.8 and 2.0.

### 4.7. PARN-002Z RT2 ProfilerTM PCR Array Rat Drug Metabolism

Analysis of Gene expression was performed using Rat Drug Metabolism RT^2^ Profiler PCR Array (Cat No PARN-002Z) (Qiagen, USA). The array contains 84 genes critical to the metabolism of drugs, toxic chemicals, hormones, and micronutrients important to pharmacology, endocrinology, and food science. Drug metabolism is also often implicated in many disease states, including cancer, intoxification, addiction, and metabolic diseases. The genes encoding enzymes that are important for drug transport (such as metallothioneins and P-glycoproteins), phase I metabolism (specifically the P450 family), and phase II metabolism (such as transferases and hydrolases) are represented on the array. Using real-time PCR, the expression of a focused panel of genes related to drug metabolism was analysed with this array. The genes involved in each pathway include

Drug Transporters

(Metallothioneins; *Mt3*)

P-Glycoprotein Family Members; *Abcb1a (Mdr1)*, *Abcb1b*, *Abcb4*, *Abcc1 (Mrp1)*, *Gpi*.

Phase I Metabolising Enzymes

Cytochrome P450s; *Cyp17a1*, *Cyp19a1*, *Cyp1a1*, *Cyp1a2*, *Cyp1b1*, *Cyp27b1*, *Cyp2b15*, *Cyp2b3*, *Cyp2c13*, *Cyp2c6v1*, *Cyp2c7*, *Cyp2e1*, *Cyp3a23/3a1*, *Cyp4b1.*

Phase II Metabolising Enzymes

Carboxylesterases; *Ces1e*, *Ces2c* (QuantiNova Symbol: LOC100365112).

Decarboxylases; *Gad1*, *Gad2*.

Dehydrogenases; *Adh1*, *Adh4*, *Alad*, *Aldh1a1*, *Hsd17b1*, *Hsd17b2*, *Hsd17b3*.

Glutathione Peroxidases (*GPx*); *Gpx1*, *Gpx2*, *Gpx3*, *Gpx4*, *Gpx5*, *Gsta1*, *Gsta4*, *Gstm1 (Mgst1)*, *Gstm2*, *Gstm3*, *Gstm4*, *Gstm5* (*QuantiNova Symbol*: *Gstm3l*), *Gstp1*, *Gstt1*, *Lpo*, *Mpo*.

Hydrolases; *Ephx1*, *Faah*, *Fbp1*.

Kinases; *Hk2*, *Pklr*, *Pkm*.

Lipoxygenases; *Alox15*, *Alox5*, *Apoe*.

Oxidoreductases; *Blvra*, *Blvrb*, *Cyb5r3*, *Gpx1*, *Gpx2*, *Gsr*, *Mthfr*, *Nos2 (Nos2a*, *iNos)*, *Nos3 (eNOS)*, *Nqo1*, *Srd5a1*, *Xdh (Srd5a2).*

Paraoxonases; *Pon1*, *Pon2*, *Pon3.*

Glutathione S-Transferases; *Chst1*, *Gsta1*, *Gstm2*, *Gstm3*, *Gstm5 (QuantiNova Symbol: Gstm3l)*, *Gstp1*, *Gstt1*, *Mgst1*, *Mgst2*, *Mgst3.*

Transferases; *Nat1*

Other Phase II Metabolising Genes: *Comt*, *Ggt1.*

Other Drug Metabolism Genes: *Ahr*, *Aoc1*, *Arnt*, *Asna1*, *Gckr*, *Marcks*, *Smarcal1*, *Snn*

For the RT^2^ Profiler PCR Array, an RT2 PreAMP cDNA Synthesis Kit was used to convert RNA elute (500 ng) to cDNA. cDNA was mixed with RT2 qPCR Master Mix and then pippetted by 100 µL into each PCR array well. The samples were measured using Real Time PCR Instrument (StepOnePlus, ABI Biosystem, Foster City, CA, USA). RT2 Profiler PCR Data Analysis Software was used to analyse data. For the data normalisation step, Housekeeping Genes (*B2M*, *Hprt1*, *Ldha*, *Rplp1*) were used. Fold regulation indicates fold-change results in a biologically meaningful way. A fold-change of more than one shows upregulation, whereas values lesser than one show downregulation. Regarding the fold regulation calculation, gene expression fold regulation was computed automatically with the Qiagen RT^2^ Profiler™ PCR Array Data Analysis software using the ΔΔCt method, where positive values indicate upregulation (2^–ΔΔCt^) and negative values indicate downregulation [–1/(2^–ΔΔCt^)]. For data quality control, only genes with Ct < 35, a single melting-curve peak, and stable housekeeping gene expression (ΔCt < 0.5 across groups) were included for analysis.

### 4.8. Statistical Analysis

All data for in vivo animal study were analysed using one-way analysis of variance (ANOVA) and Dunnett’s post hoc test. The level of significance was evaluated at a *p* value less than 0.05 (*p* < 0.05). For gene expression results, the results were analysed using Student’s *t*-test on the replicate (2^–ΔΔCt^) values for each gene in the groups. The cut-off points were >2 fold-change and *p*-value < 0.05. Graphpad Prism 8 was used for statistical analysis.

## 5. Conclusions

The administration of *F. deltoidea* aqueous extract (2000 mg/kg body weight) from gestation days 6 to 20 did not result in maternal toxicity or foetal skeletal malformations in Sprague Dawley rats. However, further investigation into hepatic gene expression changes during pregnancy is recommended to gain a deeper understanding of the physiological effects of *F. deltoidea* extract, which will help establish its safety profile.

## Figures and Tables

**Table 1:** Flavonoids identified in *F. deltoidea* var*. kunstleri* extract analysed using HPLC coupled to tandem mass spectrometry, as illustrated in Figure 1.

Peak	*t*_R_ (min)	λ_max_	Compound	[M−H]^−^ (*m*/*z*)	MS^2^ Fragment Ions (*m*/*z*)
1	6.6	270	(+)-Gallocatechin	305	261, 221, 179
2	11.4	275	(–)-Epigallocatechin	305	261, 221, 179
3	12.5	280	(+)-Catechin	289	245, 205, 179
4	12.7	280	(Epi)catechin-(Epi)afzelechin	561	435, 289, 273
5	14.2	280	(Epi)catechin-(Epi)afzelechin	561	435, 289, 273
6	15.1	275	(Epi)catechin-(Epi)afzelechin-(Epi)afzelechin	833	561, 543, 289
7	15.9	280	(Epi)catechin-(Epi)afzelechin	561	435, 289, 271
8	18.5	275	(Epi)catechin-(Epi)afzelechin-(Epi)afzelechin	833	561, 289, 271
9	19.4	280	(–)-Epicatechin	289	245, 205, 179
10	21.8	350	Luteolin-6-8-*C*-diglucoside (Lucenin-2)	609	519, 489, 399
11	25.7	340	Apigenin-6, 8-*C*-diglucoside (Vicenin-2)	593	503, 473, 353
12	27.0	345	Luteolin-6-*C*-hexosyl-8-*C*-pentoside	579	489, 459, 399
13	27.8	345	Luteolin-6-*C*-glucosyl-8-*C*-arabinoside	579	489, 459, 399
14	29.6	335	Apigenin-6-*C*-arabinosyl-8-*C*-glucoside (Isoschaftoside)	563	503, 473, 443
15	30.4	345	Luteolin-6-*C*-arabinosyl-8-*C*-glucoside	579	489, 459, 399
16	30.7	335	Apigenin-6-*C*-glucoside-8-*C*-arabinoside (Schaftoside)	563	503, 473, 443
17	31.4	335	Luteolin-8-*C*-glucoside (Orientin)	447	369, 357, 327
18	31.9	320	Apigenin-6-*C*-pentosyl-8-*C*-glucoside	563	473, 443, 353
19	33.0	310	4-*p*-Coumaroylquinic acid	337	191, 173, 163
20	35.5	335	Apigenin-8-*C*-glucoside (Vitexin)	431	413, 341, 311
21	36.5	335	Apigenin-6-*C*-glucosyl-8-*C*-pentoside	563	473, 443, 353
22	37.5	335	Apigenin-6,8*-C-*dipentoside	533	515, 473, 443
23	38.5	335	Apigenin-6,8*-C-*dipentoside	533	515, 473, 443
24	39.6	335	Apigenin-6-*C*-glucoside (Isovitexin)	431	413, 341, 311
25	42.1	335	Apigenin-6,8*-C-*dipentoside	533	515, 473, 443
26	44.7	335	Apigenin-6,8*-C-*dipentoside	533	515, 473, 443

**Table 2:** Relative organ weight of pregnant rats treated with *F. deltoidea* var*. kunstleri* aqueous extract at different concentrations during gestation period.

Organs	Relative Organ Weight (Organ Weight/Body Weight at Necropsy)				
	Control	250	500	1000	2000
Liver	3.03 ± 0.320	3.34 ± 0.196 *	3.30 ± 0.310 *	3.20 ± 0.222 *	3.42 ± 0.267 *
Kidney (R)	0.20 ± 0.014	0.21 ± 0.005	0.22 ± 0.013	0.21 ± 0.013	0.22 ± 0.024
Kidney (L)	0.20 ± 0.008	0.21 ± 0.008	0.21 ± 0.018	0.21 ± 0.019	0.22 ± 0.031
Heart	0.22 ± 0.018	0.24 ± 0.013	0.22 ± 0.021	0.23 ± 0.020	0.21 ± 0.006
Lung	0.35 ± 0.031	0.33 ± 0.021	0.37 ± 0.073	0.32 ± 0.048	0.36 ± 0.042
Ovary (right)	0.016 ± 0.005	0.015 ± 0.004	0.018 ± 0.007	0.016 ± 0.006	0.016 ± 0.003
Ovary (left)	0.017 ± 0.004	0.016 ± 0.007	0.020 ± 0.006	0.016 ± 0.007	0.017 ± 0.004

Data are shown as mean ± SD (*n* = 5) and analysed by ANOVA. * Significant difference with *p* < 0.05.

**Table 3:** Modified genes in maternal liver after treatment with different concentrations of *F. deltoidea* var. *kunstleri* aqueous extract.

		*F. deltoidea* Aqueous Extract (mg/kg Body Weight)					
Gene Symbol	Gene Name	250		500		2000	
		Fold Regulation	*p*-Value	Fold Regulation	*p*-Value	Fold Regulation	*p*-Value
***Abcb1a***	ATP-binding cassette, sub-family B (MDR/TAP), member 1A	−61.45	0.0047	−10.16	0.0423		
***Abcb1b***	ATP-binding cassette, sub-family B (MDR/TAP), member 1B	−2.59	0.00002				
***Abcb4***	ATP binding cassette subfamily B member 4	3.94	0.0065			−76.40	0.0034
***Ahr***	aryl hydrocarbon receptor	5.20	0.0238				
***Arnt***	aryl hydrocarbon receptor nuclear translocator	−52.80	0.0240			−40.75	0.0175
***Blvra***	biliverdin reductase A					−12.00	0.0332
***Ces2c***	carboxylesterase 2C					−49.43	0.0481
***Cyb5r3***	cytochrome b5 reductase 3	−116.62	0.0300				
***Cyp19a1***	cytochrome P450 family 19 subfamily A member 1	−2.59	0.00002				
***Cyp27b1***	cytochrome P450 family 27 subfamily B member 1	−5.95	0.0101			−36.33	0.0067
***Cyp4b1***	cytochrome P450 family 4 subfamily B member 1					−52.57	0.0086
***Faah***	fatty acid amide hydrolase	−158.27	0.0050			−166.92	0.0051
***Gad1***	glutamate decarboxylase 1	−2.59	0.00002				
***Gckr***	glucokinase regulator	7.97	0.0062				
***Gpi***	glucose-6-phosphate isomerase					−14.07	0.0450
***Gpx3***	glutathione peroxidase 3	−231.63	0.0044				
***Gpx5***	glutathione peroxidase 5			−32.81	0.0413	−56.78	0.0217
***Gstm1***	glutathione S-transferase mu 1	3.27	0.0322				
***Gstt1***	glutathione S-transferase theta 1	−796.33	0.0405	−28.04	0.0476	−162.61	0.0449
***Nat1***	N-acetyltransferase 1	5.36	0.0170				
***Pkm***	Pyruvate Kinase M1/2)			−54.88	0.0110	−68.91	0.0115
***Snn***	stannin	−2.59	0.00002				

Note: Fold regulation values are reported exactly as generated with the RT^2^ Profiler PCR Array software version 3.5. Negative values indicate downregulation relative to the control and may appear large due to the software’s log-based transformation. *p*-values are shown as exported from the original output file and follow the fixed decimal formatting produced by the analysis system.

**Table 4:** Percentages of the incidences of abnormalities in the foetal skeletal of SD rats orally treated with *F. deltoidea* var*. kunstleri* aqueous extract (0, 250, 500, 1000, and 2000 mg/kg/day) on gestation days 6 to 20. Values are % of foetuses showing abnormalities and litter means (percentage of affected foetuses per litter; *n* = 5/group). Comparisons were made via one-way analysis of variance (ANOVA) and Dunnett’s post hoc test. Proportions different (*p* < 0.05) from the control group are indicated with an asterisk (*). Ossif. Center: ossification centre; Misshap: misshapened; Incpl.ossif: incomplete ossification; ad ossif: additional ossification.

Treatment	*F. deltoidea* Aqueous Extract (mg/kg Body Weight/Day)				
	0	250	500	1000	2000
Foetuses examined (n)	30	30	30	30	30
Litters examined (n)	5	5	5	5	5
Percentage of foetuses showing anomalies (%) and litters affected (%)					
SKULL					
Os.Parietale (incpl.ossif.)	13.33 (55.56)	12.67 (52.78)	14.67 (61.11)	17.33 (72.22)	16 (66.67)
Os.Frontale (incpl.ossif.)	1.33 (5.56)	2 (8.33)	8.67 * (36.11)	12.67 * (52.78)	6.67 * (27.78)
Os.Occipitale (incpl.ossif.)	2.67 (11.11)	7.33 * (30.56)	6.67 (27.78)	4.67 (19.44)	6.67 (27.78)
Os. Interparietale (ad.ossif.)	7.33 (30.56)	8.67 (36.11)	15.33 * (63.89)	16.67 * (69.44)	17.33 * (72.22)
Os hyoid (absent) (incpl.ossif)	0.67 (2.78)	8.67 * (36.11)	3.33 (13.89)	10 * (41.67)	2 (8.33)
	4.67 (19.44)	2 (8.33)	2.67 (11.11)	1.33 (5.56)	3.33 (13.89)
Proc. Jugalis maxilla (incpl.ossif.)	2 (8.33)	3.33 (13.89)	3.33 (13.89)	4.67 (19.44)	2 (8.33)
Os. Zygomatic (incpl.ossif.)	3.33 (13.89)	4 (16.67)	4.67 (19.44)	1.33 (5.56)	5.33 (22.22)
STERNUM					
All stenerbrae (split)	0	0	0	0	0
(misaligned)	0	0	0.67 (2.78)	0	0
Sternebra 1 (split)	0	0	0	0	0
(incpl.ossif.)	0	0.67 (2.78)	0	0	0
(misaligned)	0	0	0	0	0
Sternebra 2 (misshap.)	0	0	0	0	0
(smaller)	0	0	0.67 (2.78)	0	0
(incpl.ossif.)	0	0.67 (2.78)	0	0	0.67 (2.78)
(misaligned)	0	0	0	1.33 (5.56)	0.67 (2.78)
Sternebra 3 (misshap.)	0	0	0	0	0
(smaller)	0	0	0	0	0
(incpl.ossif.)	0.67 (2.78)	0	0	0	2 (8.33)
(misaligned)	0	0	0.67 (2.78)	2 (8.33)	2.67 (11.11)
Sternebra 4 (misshap.)	0	0	0	0	0
(incpl.ossif)	0.67 (2.78)	0	0	0	0
(misaligned)	0	0	0.67 (2.78)	2 (8.33)	2 (8.33)
Sternebra 5 (misshap.)	0.67 (2.78)	0	0	4 (16.67)	4 (16.67)
(smaller)	0	0	0	0	0
(incpl.ossif.)	3.33 (13.89)	5.33 (22.22)	0.67 (2.78)	4.67 (19.44)	5.33 (22.22)
(absent)	0.67 (2.78)	0.67 (2.78)	0	0.67 (2.78)	1.33 (5.56)
(misaligned)	0.67 (2.78)	0	0.67 (2.78)	1.33 (5.56)	0.67 (2.78)
Xiphisternum (split)	0	0	0	0	0
(incpl.ossif.)	1.33 (5.56)	1.33 (5.56)	0.67 (2.78)	0	8 * (33.33)
(absent)	0	0	0	1.33 (5.56)	0
RIBS					
(fused)	0	0	0	0	0
(wavy)	4 (16.67)	2.67 (11.11)	4.67 (19.44)	2.67 (11.11)	6.67 (27.78)
(incpl.ossif)	1.33 (5.56)	1.33 (5.56)	3.33 (13.89)	1.33 (5.56)	2 (8.33)
13th rib (short)	0	0	0	0	0
Supernumery ribs (short)	0	0	0	0	0
(both sides)	0	0	0	0	0
(one sides)	0	0	0	0	0
14th Rib (rudimentary)	0	0	0	0	0
(both sides)	0.67 (2.78)	0.67 (2.78)	1.33 (5.56)	0.67 (2.78)	0
(one side)	0.67 (2.78)	0.67 (2.78)	0	1.33 (5.56)	0.67 (2.78)
VERTEBRAL COLUMN					
Atlas (misshap)	0	0	0	0	0.67 (2.78)
(incpl.ossif.)	0.67 (2.78)	1.33 (5.56)	2.67 (11.11)	0.67 (2.78)	2 (8.33)
Thoracic verto.c. (dumbbell) (bipartite) (hemicentric) (split)	4 (16.67)	2.67 (11.11)	11.33 * (47.22)	11.3 * (47.22)	8.67 (36.11)
	0	0	0	0	0.67 (2.78)
	0	0	0	0	0
	0	0	0	4 (16.67)	4 (16.67)
Lumbar vert (dumbbell)	0	0	0.67 (2.78)	0	0
(bipartite)	0	0	0	1.33 (5.56)	0.67 (2.78)
(split)	0	0	0		
FORELIMBS					
Fingers (poorly ossified)	19.33 (80.56)	18.67 (77.78)	16.67 (69.44)	18 (75.00)	18 (75.00)
Os humerus (incpl.ossif.)	0	2 (8.33)	2 (8.33)	0	0.67 (2.78)
HINDLIMBS					
Os femur (misshap.) (incpl.ossif)	1.33 (5.56)	7.33 * (30.56)	5.33 (22.22)	7.33 * (30.56)	8 * (33.33)
	0	2 (8.33)	0	0	0

## Data Availability

The data that support the findings of this study are available from the corresponding author upon reasonable request. The data are not publicly available due to [ethical and confidentiality restriction].
